# Prognostic impact of CRAFITY score in hepatocellular carcinoma patients treated with immune checkpoint inhibitors

**DOI:** 10.1016/j.isci.2025.113976

**Published:** 2025-11-10

**Authors:** Lilong Zhang, Yuefeng Zhang, Kunpeng Wang, Jiarui Feng, Chen Chen, Xinfei Liu, Weixing Wang

**Affiliations:** 1 Department of General Surgery, Renmin Hospital of Wuhan University, Wuhan, Hubei 430060, China; 2 Hubei Key Laboratory of Digestive System Disease, Renmin Hospital of Wuhan University, Wuhan, Hubei 430060, China; 3 General Surgery Laboratory, Renmin Hospital of Wuhan University, Wuhan, Hubei 430060, China; 4 Central Laboratory, Renmin Hospital of Wuhan University, Wuhan, Hubei 430060, China; 5 Department of Hematology Renmin Hospital of Wuhan University, Wuhan, Hubei 430060, China

**Keywords:** Therapeutics, Cancer

## Abstract

Immune checkpoint inhibitors (ICIs) have transformed the treatment landscape of hepatocellular carcinoma (HCC), yet identifying patients most likely to benefit remains challenging. We evaluated the prognostic significance of the C-reactive protein and alpha-fetoprotein in immunotherapy (CRAFITY) score, a composite index integrating C-reactive protein and alpha-fetoprotein, in HCC treated with ICIs. A systematic review and meta-analysis of 17 studies comprising 3,730 patients demonstrated that higher baseline CRAFITY scores were consistently associated with shorter overall and progression-free survival, as well as lower response and disease control rates. Complementing these findings, a retrospective analysis of 129 ICI-treated patients at our institution confirmed that elevated CRAFITY scores predicted significantly poorer survival outcomes. These results highlight the CRAFITY score as a simple, readily available biomarker that captures systemic inflammation and tumor burden, enabling more precise prognostic stratification and potentially guiding therapeutic decision-making in patients receiving immunotherapy for advanced HCC.

## Highlights

• Higher CRAFITY scores predict shorter survival in ICI-treated HCC patients
• Meta-analysis of 17 studies confirms robust prognostic value of CRAFITY score
• CRAFITY may guide risk stratification and treatment decisions in advanced HCC

## Graphical abstract

**Figure:**
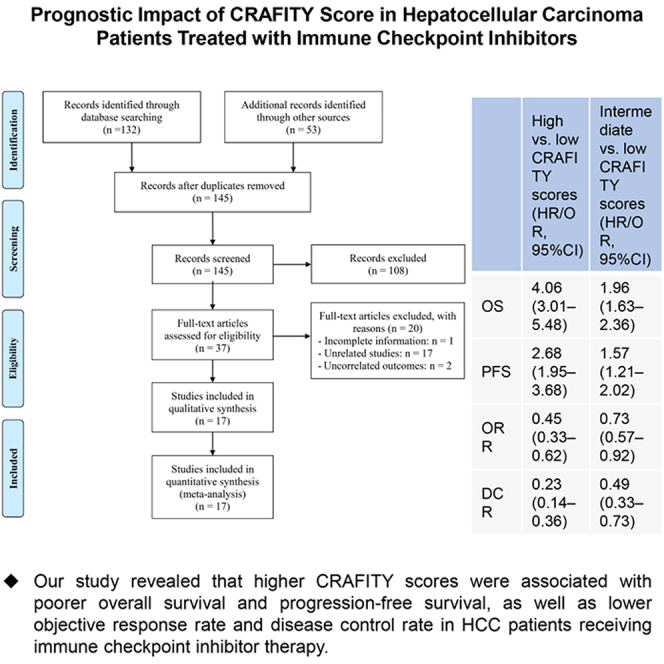

## Introduction

Hepatocellular carcinoma (HCC) remains one of the most lethal malignancies worldwide, largely attributable to the paucity of effective therapeutic options for patients diagnosed at advanced stages.^1^ Sorafenib, a multikinase inhibitor, was the first systemic agent approved for unresectable HCC, as demonstrated by the pivotal SHARP and Asia-Pacific trials.^2^^,^^3^ However, therapeutic advances have shifted the standard of care with the advent of immune-based regimens. Notably, the IMbrave150 study established the combination of atezolizumab and bevacizumab as the preferred first-line treatment, showing a substantial improvement in clinical outcomes, including an objective response rate (ORR) of 29.8% and an extension in median overall survival (OS) of 5.8 months compared to sorafenib monotherapy.^4^ This study marked a paradigm shift from antiangiogenic monotherapies to immunotherapy-centered combination strategies.

Despite these advancements, patient responses to immune checkpoint inhibitors (ICIs), particularly those targeting the programmed cell death protein 1 (PD-1) pathway, remain heterogeneous, with up to one-third of patients exhibiting primary resistance.^5^ Such variability underscores the urgent need for reliable and accessible predictive biomarkers to guide immunotherapy selection and optimize clinical outcomes in HCC.^6^

To address this unmet need, Scheiner et al.^7^ proposed the CRAFITY score (C-reactive protein and alpha-fetoprotein in immunotherapy), a simplified prognostic tool combining serum levels of C-reactive protein (CRP) and alpha-fetoprotein (AFP). In this model, elevated CRP (>1 mg/dL) and AFP (>100 ng/mL) are each assigned one point, stratifying patients into three risk categories: score 0 (neither marker elevated), score 1 (only one elevated), and score 2 (both elevated). While initial studies suggested a prognostic association with immunotherapy outcomes, subsequent investigations by Huang et al.,^8^ Kaneko et al.,^9^ and Zhang et al.^10^ reported inconsistent findings, particularly regarding the relationship between baseline CRAFITY scores and progression-free survival (PFS) in ICI-treated HCC patients.

The CRAFITY score has been proposed as a prognostic biomarker in HCC, but existing studies have reported inconsistent findings and have largely lacked systematic evidence synthesis or external validation. To date, no meta-analysis has been performed to comprehensively assess the relationship between the CRAFITY score and clinical outcomes in HCC patients undergoing ICIs. The present study addresses this gap by integrating available evidence through a systematic meta-analysis and subsequently validating the results in an independent single-center cohort. In addition, our study expands upon prior research by evaluating not only OS but also radiologic-based endpoints, including PFS, ORR, and disease control rate (DCR). This combined approach strengthens the prognostic value of the CRAFITY score and provides insights into its potential utility for risk stratification and treatment decision-making in patients with HCC receiving ICI therapy.

## Results

### Search results and study characteristics

**Figure 1:**
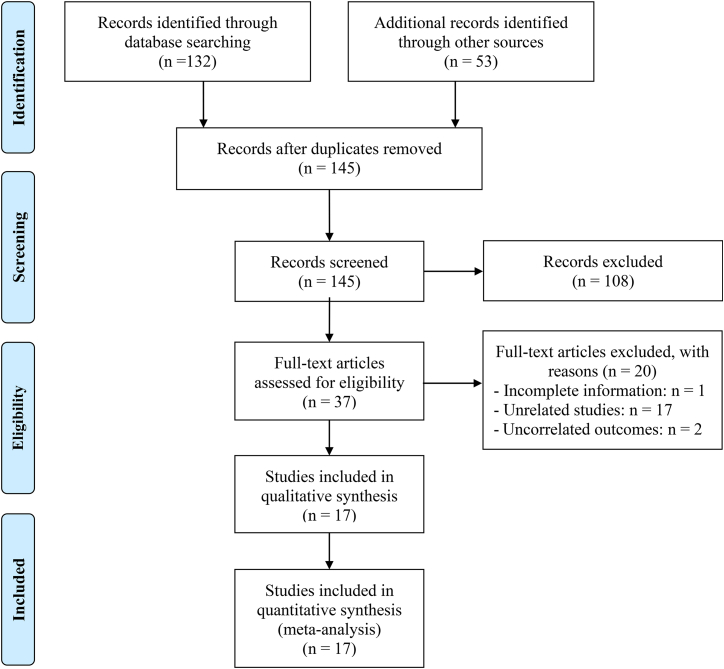
The flow diagram of identifying eligible studies

A comprehensive literature query, combining database exploration with manual examination of bibliographic references, initially identified 185 candidate publications. Upon the elimination of 40 redundant entries, 108 articles were discarded based on title and abstract appraisal due to non-conformity with established inclusion parameters. Subsequent full-text scrutiny of 37 shortlisted manuscripts led to the exclusion of 20 additional studies that failed to fulfill the eligibility benchmarks. Ultimately, 17 investigations satisfied all selection criteria and were incorporated into the final meta-analysis^7^^,^^8^^,^^9^^,^^10^^,^^11^^,^^12^^,^^13^^,^^14^^,^^15^^,^^16^^,^^17^^,^^18^^,^^19^^,^^20^^,^^21^^,^^22^^,^^23^ (Figure 1).

A detailed summary of the principal attributes of the studies incorporated in this analysis is presented in Table 1. Cumulatively, 3,730 participants were assessed across the included investigations, with individual study cohorts varying between 45 and 302 subjects. Geographically, the majority of research was conducted in China (*n* = 12), while four studies originated from Japan. All eligible studies utilized a retrospective methodological framework. According to the Newcastle-Ottawa Scale (NOS) assessment, the methodological quality was deemed robust, with scores spanning from 6 to 8, suggesting minimal susceptibility to bias (Table 1).

**Table 1:** Main characteristics of the studies included

Study	During the study	Country	Number	Age	Gender	Treatment	Treatment line	NOS score
Teng et al.^19^	09/2020–01/2022	China	89	61.3 (56.4–67.8)^a^	75/14	atezolizumab + bevacizumab	–	7
Hatanaka et al.^12^	09/2020–11/2021	Japan	297	73.0 (68.0–78.0)^a^	243/54	atezolizumab + bevacizumab	front (169)/later (128) line	8
Zhou et al.^23^	01/2018–12/2021	China	157	58.5±10.5^b^	117/40	TACE + apatinib + camrelizumab	–	8
Zhang et al.^10^	01/2019–06/2022	China	70	53.8± 10.4^b^	58/12	TACE + TKIs + PD-1 inhibitors	–	6
Wu et al.^21^	10/2018–08/2023	China	228	55.5 (48.8–62.0)^a^	209/19	pembrolizumab + lenvatinib	–	7
Ueno et al.^20^	10/2020–09/2022	Japan	302	73.0 (66.0–79.0)^a^	238/64	atezolizumab + bevacizumab	–	7
Yin et al.^22^	01/2018–01/2022	China	192	57.5 (50.0, 64.0)^a^	161/31	lenvatinib + (camrelizumab or sintilimab)	1^st^ (147)/later (45) line	7
Lieb et al.^16^	–	Germany	45	68.0 ± 11.0^b^	40/5	atezolizumab + bevacizumab	–	6
Ouyang et al. (T)^18^	03/2020–02/2022	China	150	51/99^d^	132/18	lenvatinib + PD-1 inhibitors	–	7
Ouyang et al. (V)^18^	08/2018–11/2021	China	115	38/77^d^	102/13	lenvatinib + PD-1 inhibitors	–	7
Huang et al.^8^	01/2019–03/2023	China	133	56.0 ± 11.0^b^	118/15	(TACE or HAIC) + TKIs + PD-(L)1 inhibitors	–	7
Guan et al.^11^	01/2019–12/2019	China	171	53.0 (43.0–58.0)^a^	147/24	(TACE or HAIC) + ICIs	–	7
Hsu et al.^13^	05/2017–03/2022	China	110	64.5 (54.6–72.4)^a^	94/16	nivolumab or pembrolizumab	1^st^ (35)/2^nd^ (53)/3^rd^ (16) /4^th^ (6) line	8
Hu et al.^14^	11/2019–11/2022	China	85	61.0 ± 9.9^b^	74/11	TACE + MTAs + PD-(L)1 inhibitors	–	7
Kang et al.^15^	01/2019–06/2023	China	158	55.2 ± 10.8^b^	140/18	TACE + MTAs + PD-(L)1 inhibitors	–	7
Ohama et al.^17^	09/2020–01/2023	Japan	719	74.0 (69.0–80.0)^a^	577/142	atezolizumab + bevacizumab	1^st^ (487)/2^nd^ (158)/3^rd^ (47)/4^th^ (20)/5^th^ (7)	7
Kaneko et al.^9^	11/2020–03/2023	Japan	213	74.0 (34.0–91.0)^c^	183/30	atezolizumab + bevacizumab	–	8
Scheiner et al.^7^	07/2015–12/2020	Austria, Germany, Italy, and Switzerland	292	–	236/56	ICIs	–	8

Abbreviations: HR, hazard ratio; CI, confidence interval.

ICIs, immune checkpoint inhibitors; TACE, transcatheter arterial chemoembolization; TKIs, tyrosine kinase inhibitors; PD-(L)1, programmed cell death ligand 1; HAIC, hepatic arterial infusion chemotherapy; MTAs, molecular targeted agents; T, training queue; V, validation queue.

a Median (first quartile-third quartile).

b Mean ± standard deviation.

c Median (range).

d >60/<60.

### Baseline CRAFITY and OS

**Figure 2:**
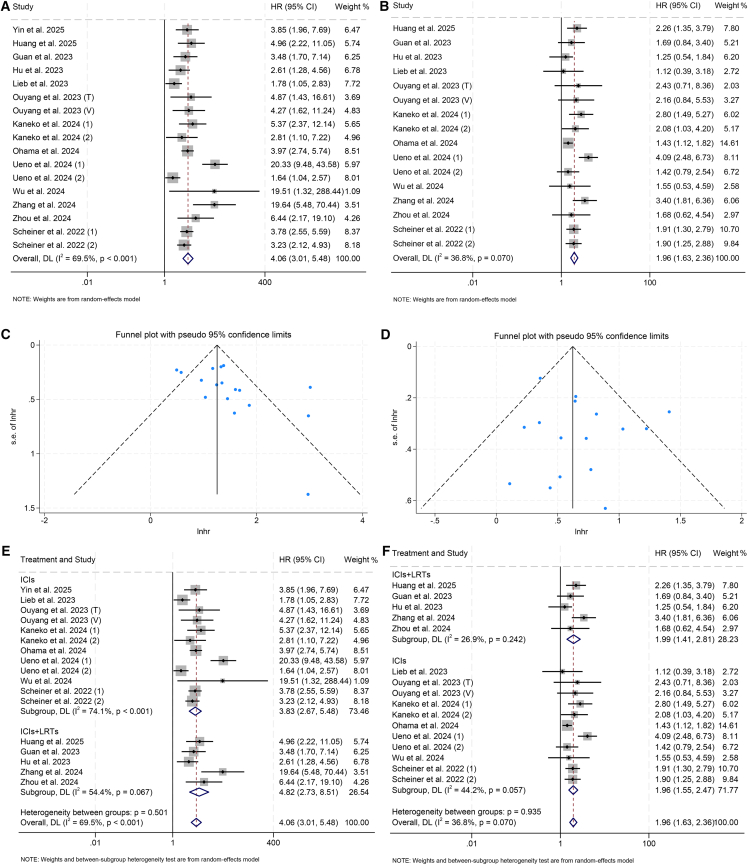
Baseline CRAFITY classification predicts OS in HCC patients treated with ICIs (A and B) Forest plot analyses of the association between baseline CRAFITY classification and OS. (A) High vs. low CRAFITY scores. (B) Intermediate vs. low CRAFITY scores. (C and D) Funnel plots assessing publication bias for the relationship between CRAFITY classification and OS. (C) High vs. low CRAFITY scores. (D) Intermediate vs. low CRAFITY scores. (E and F) Stratified analyses according to treatment modality evaluating the prognostic impact of CRAFITY classification on OS. (E) High vs. low CRAFITY scores. (F) Intermediate vs. low CRAFITY scores. Abbreviations: HR, hazard ratio; CI, confidence interval; LRTs, locoregional therapies.

Sixteen studies with 3,229 patients were included to assess the relationship between CRAFITY and OS in ICI-treated HCC patients. Pooled analysis demonstrated that higher CRAFITY scores were strongly associated with significantly shorter OS, with a stepwise survival gradient observed from low to intermediate to high CRAFITY groups (high vs. low: hazard ratio [HR] = 4.06, 95% confidence interval [CI]: 3.01–5.48, *p* < 0.001, Figure 2A; intermediate vs. low: HR = 1.96, 95% CI: 1.63–2.36, *p* < 0.001; Figure 2B). Similar associations were confirmed in studies that analyzed CRAFITY as a binary variable (HR: 2.44, 95% CI: 1.25–4.74, *p* = 0.009; Figure S1). Statistical heterogeneity across the included studies was confirmed by both Cochran’s Q statistic and elevated I^2^ metrics, thereby justifying the application of a random-effects modeling framework.

Sensitivity analysis was conducted to assess the robustness of the pooled results by sequentially omitting each individual study. Throughout the process, the overall estimate for OS remained consistent, indicating the stability of the findings (Figures S2A and S2B). The funnel plot appeared approximately symmetrical, suggesting no significant publication bias (Figures 2C and 2D).

Subgroup analyses demonstrated that the prognostic value of the CRAFITY score was consistently maintained across both univariate and multivariate Cox regression models, underscoring its independent predictive utility (Figures S2C and S2D). Importantly, this association remained robust regardless of whether ICI-based therapy was administered as monotherapy or in combination with locoregional therapies (LRTs) (Figures 2E and 2F). Moreover, considering the heterogeneity in variables adjusted for across multivariate models, we additionally synthesized outcomes based on univariate analyses, which confirmed the persistent association between elevated CRAFITY scores and inferior OS (Figures S2E and S2F). From a clinical perspective, these findings highlight that systemic inflammation and impaired liver function—both reflected in the CRAFITY score—represent pivotal determinants of long-term survival in HCC patients undergoing ICI-based therapy.

### Baseline CRAFITY and PFS

**Figure 3:**
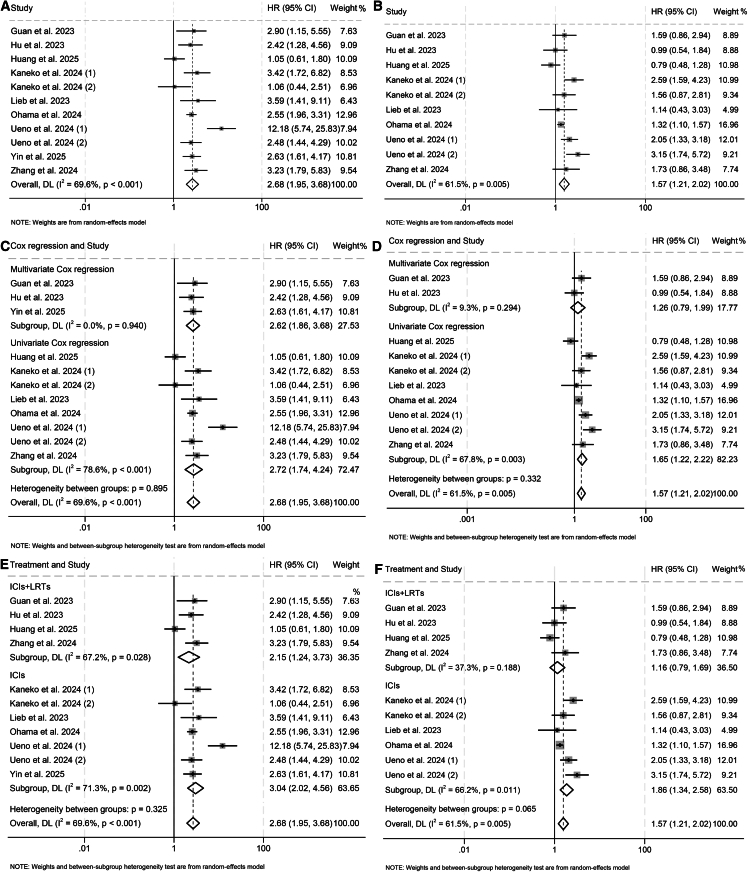
Baseline CRAFITY classification is associated with PFS in HCC patients receiving ICIs (A and B) Forest plot analyses of the association between baseline CRAFITY classification and PFS. (A) High vs. low CRAFITY scores. (B) Intermediate vs. low CRAFITY scores. (C and D) Stratified analyses using Cox proportional hazards models evaluating the prognostic impact of pretreatment CRAFITY classification on PFS. (C) High vs. low CRAFITY scores. (D) Intermediate vs. low CRAFITY scores. (E and F) Stratified analyses according to treatment modality evaluating the prognostic impact of CRAFITY classification on PFS. (E) High vs. low CRAFITY scores. (F) Intermediate vs. low CRAFITY scores. Abbreviations: HR, hazard ratio; CI, confidence interval; LRTs, locoregional therapies.

Twelve studies including 2,287 patients indicated that higher baseline CRAFITY scores predicted shorter PFS. A clear gradient effect was again observed, with patients in the high-score group showing the poorest outcomes (high vs. low: HR = 2.68, 95% CI: 1.95–3.68; *p* < 0.001, Figure 3A; intermediate vs. low: HR = 1.57, 95% CI: 1.21–2.02, *p* < 0.001; Figure 3B). Substantial inter-study variability was confirmed through both Cochran’s Q test and elevated I^2^ statistics, thereby necessitating the adoption of a random-effects modeling approach to account for heterogeneity.

Furthermore, in three independent investigations where the CRAFITY score was treated as a dichotomous parameter, meta-analytic integration revealed a significant correlation between elevated CRAFITY levels and inferior PFS (I^2^ = 0%, *p* = 0.729; HR: 1.82, 95% CI: 1.46–2.27, *p* < 0.001, Figure S3).

To evaluate the reliability of the pooled estimates, sensitivity testing was performed by stepwise exclusion of individual studies. Across all iterations, the summary HR for PFS remained stable, reinforcing the credibility of the findings (Figures S4A and S4B). Visual inspection of the funnel plot did not reveal conspicuous asymmetry, implying the absence of significant publication bias (Figures S4C and S4D). This observation was further corroborated by Begg’s (high vs. low, *p* = 0.436; intermediate vs. low, *p* = 1.000) and Egger’s test (high vs. low, *p* = 0.677; intermediate vs. low, *p* = 0.473), which indicated no statistically significant evidence of bias.

The subgroup analysis revealed that a high CRAFITY score was consistently associated with worse PFS compared to low CRAFITY score, regardless of whether the analysis was conducted using univariate or multivariate models (Figures 3C and 3D), and irrespective of the treatment regimen (ICI therapy alone or ICI combined with LRTs) (Figures 3E and 3F). In contrast, when comparing intermediate scores with low scores, this association was only significant in univariate analysis or studies that focused solely on ICI therapy (Figures 3C–3F). In light of the variability in adjusted variables across multivariate models, we also aggregated results from univariate analyses, which further supported the ongoing correlation between higher CRAFITY scores and poorer PFS (Figures S4E and S4F).

### Baseline CRAFITY and ORR

**Figure 4:**
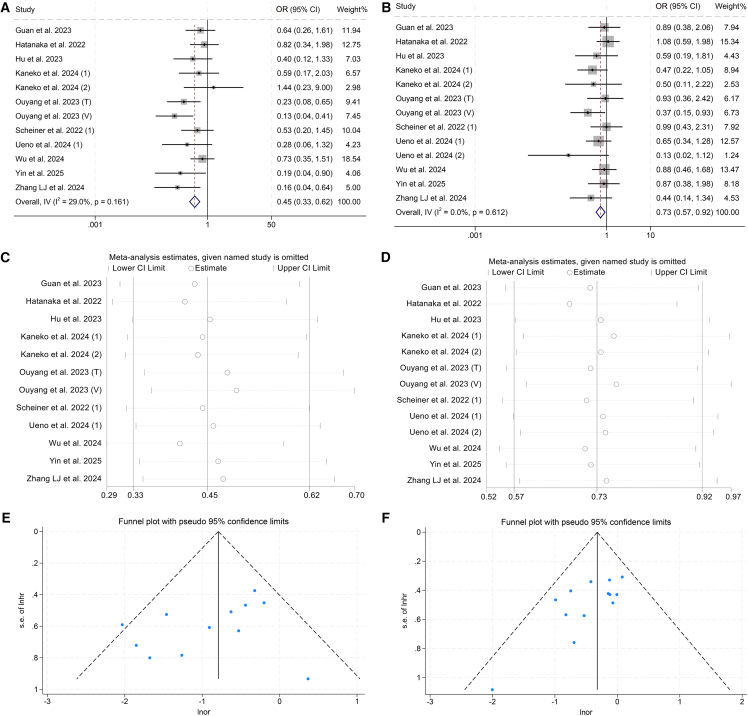
Baseline CRAFITY classification correlates with ORR in HCC patients treated with ICIs (A and B) Forest plot analyses of the association between baseline CRAFITY classification and ORR. (A) High vs. low CRAFITY scores. (B) Intermediate vs. low CRAFITY scores. (C and D) Sensitivity analyses evaluating the robustness of the association between CRAFITY classification and ORR. (C) High vs. low CRAFITY score groups. (D) Intermediate vs. low CRAFITY score groups. (E and F) Funnel plots assessing publication bias for the relationship between CRAFITY classification and ORR. (E) High vs. low CRAFITY scores. (F) Intermediate vs. low CRAFITY scores. Abbreviations: OR, odds ratio; CI, confidence interval.

A total of 10 studies with 2,115 patients were analyzed to explore the association between CRAFITY levels and ORR in HCC patients treated with ICIs. The results revealed that higher CRAFITY scores were significantly associated with lower ORR (high vs. low: odds ratio [OR] = 0.45, 95% CI: 0.33–0.62, *p* < 0.001, Figure 4A; intermediate vs. low: OR = 0.73, 95% CI: 0.57–0.92, *p* = 0.008; Figure 4B). The absence of significant between-study heterogeneity, as indicated by non-significant Cochran’s Q values in conjunction with low I^2^ estimates, justified the selection of a fixed-effects model for the meta-analytic computations.

To evaluate the stability of these associations, a leave-one-out sensitivity approach was applied, systematically removing each individual study to assess its influence on the overall effect size. The pooled ORs remained largely unaffected throughout this process, affirming the reliability of the findings (Figures 4C and 4D). Moreover, visual inspection of funnel plots did not reveal substantial asymmetry, implying the absence of major publication bias across the included studies (Figures 4E and 4F).

Analysis of the subgroups demonstrated that individuals with elevated CRAFITY scores persistently exhibited poorer ORR than those with low scores, regardless of whether they underwent ICI monotherapy or an ICI-LRT combination (Figures S5A and S5B). In comparison, the difference observed between intermediate and low scores reached statistical significance only in investigations restricted to ICI therapy (Figures S5A and S5B).

### Baseline CRAFITY and DCR

**Figure 5:**
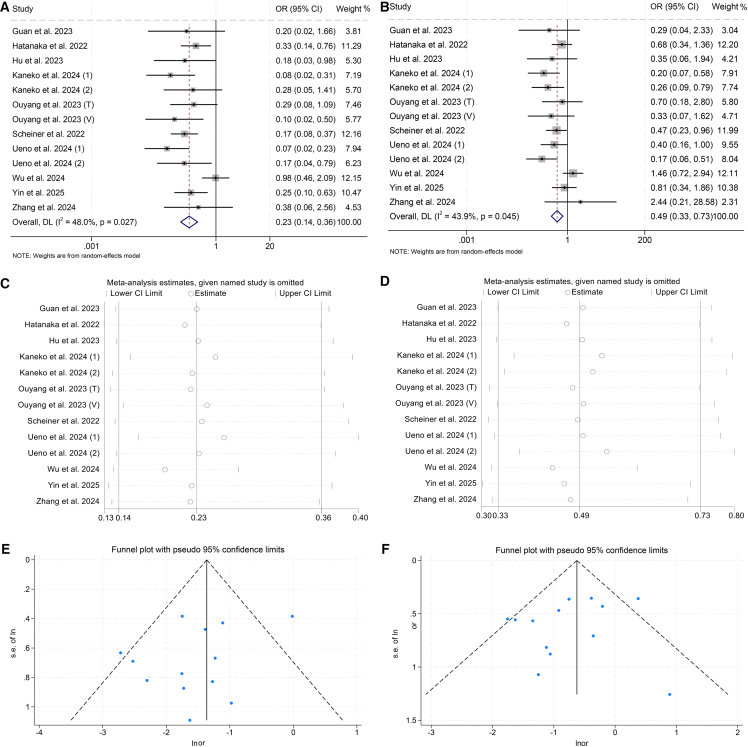
Baseline CRAFITY classification is associated with DCR in HCC patients treated with ICIs (A and B) Forest plot analyses of the association between baseline CRAFITY classification and DCR. (A) High vs. low CRAFITY scores. (B) Intermediate vs. low CRAFITY scores. (C and D) Sensitivity analyses of the association between CRAFITY classification and DCR. (C) High vs. low CRAFITY score groups. (D) Intermediate vs. low CRAFITY score groups. (E and F) Funnel plots assessing publication bias for the relationship between CRAFITY classification and DCR. (E) High vs. low CRAFITY scores. (F) Intermediate vs. low CRAFITY scores. Abbreviations: OR, odds ratio; CI, confidence interval.

Ten studies with 2,115 patients further demonstrated that higher CRAFITY scores correlated with lower DCR (high vs. low: OR = 0.23, 95% CI: 0.14–0.36, *p* < 0.001, Figure 5A; intermediate vs. low: OR = 0.49, 95% CI: 0.33–0.73, *p* < 0.001; Figure 5B). Pronounced heterogeneity across studies was evident, as supported by a significant Cochran’s Q statistic and elevated I^2^ values, which collectively warranted the implementation of a random-effects model to accommodate between-study variation.

To further validate the consistency of these findings, a leave-one-out sensitivity framework was employed. Each study was sequentially excluded to determine its impact on the aggregate effect estimates. The recalculated odds ratios showed minimal fluctuation throughout this exclusion process, supporting the robustness of the primary conclusions (Figures 5C and 5D). Additionally, symmetry in the funnel plots was preserved, providing no visual indication of substantial publication bias (Figures 5E and 5F), a result reinforced by statistical evaluation.

Subgroup evaluation revealed that patients carrying higher CRAFITY scores consistently showed inferior overall DCR compared with their low-score counterparts, independent of receiving ICI monotherapy or combined treatment with LRTs (Figures S6A and S6B). By contrast, when intermediate scores were assessed against low scores, a statistically significant difference emerged solely in analyses confined to ICI monotherapy (Figures S6A and S6B).

### Prognostic role of CRAFITY in our HCC cohort

To independently validate the prognostic relevance of the CRAFITY score, we conducted an internal analysis based on a cohort of HCC patients managed at our institution. Baseline demographic and clinical characteristics of the 129 enrolled patients are summarized in Table S1. Based on pre-treatment serum CRP and AFP levels, patients were stratified into three distinct CRAFITY categories: low (*n* = 36, 27.91%), intermediate (*n* = 54, 41.86%), and high (*n* = 39, 30.23%).

The median patient age was 56.4 years, ranging from 38.5 to 81.1 years. The study population was predominantly male, comprising 83 individuals (64.34%). In terms of functional status, 85 patients (65.89%) exhibited an ECOG performance score of 0, while the remainder (*n* = 44, 34.11%) had a score of 1. Chronic hepatitis of viral etiology was noted in 102 cases (79.07%), and underlying liver cirrhosis was reported in 96 patients (74.42%).

According to the Barcelona Clinic Liver Cancer (BCLC) staging framework, early-stage disease was identified in 7 patients (5.43%), intermediate in 37 (28.68%), and advanced in 85 individuals (65.89%). Microvascular invasion was histologically evident in 62 cases (48.06%), while multifocal tumors (≥3 lesions) were found in 60 patients (46.51%).

**Figure 6:**
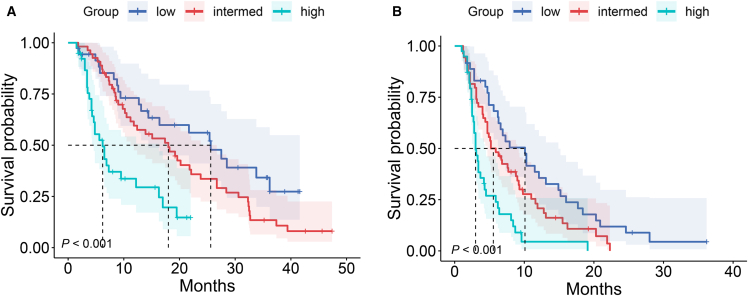
Baseline CRAFITY classification stratifies OS and PFS in patients with HCC treated with ICIs (A) Kaplan-Meier curves depicting OS based on baseline CRAFITY categories. (B) Kaplan-Meier curves depicting PFS based on baseline CRAFITY categories.

Kaplan-Meier survival curves revealed a statistically robust stratification in OS across the three CRAFITY-defined cohorts (*p* < 0.001; Figure 6A). Patients classified within the low CRAFITY category experienced the most favorable median OS, reaching 25.6 months (95% CI: 14.0–not reached), whereas individuals in the intermediate group exhibited a median survival of 18.0 months (95% CI: 11.2–26.7). In contrast, those in the high CRAFITY category had the poorest prognosis, with a median OS of just 6.2 months (95% CI: 4.6–16.3).

A similar trend was evident in PFS, where significant between-group differences were again observed (*p* < 0.001; Figure 6B). The median PFS durations were 10.1 months (95% CI: 6.3–14.9) for the CRAFITY-low group, 5.6 months (95% CI: 4.7–9.0) for the intermediate group, and only 3.0 months (95% CI: 2.7–4.3) in the high-score group.

## Discussion

The CRAFITY score, derived from circulating levels of AFP and CRP, offers a clinically feasible, cost-effective biomarker model that is easily implemented in routine practice. In our present study, elevated baseline CRAFITY scores were robustly associated with inferior survival outcomes in HCC patients undergoing ICI therapy.

Inflammation has long been recognized as a fundamental hallmark of cancer, playing a pivotal role in hepatocarcinogenesis through the dynamic remodeling of the tumor microenvironment.^24^ A range of inflammation-based indices—such as the neutrophil-to-lymphocyte ratio, monocyte-to-lymphocyte ratio, and CRP—have been linked to clinical prognosis in HCC.^25^^,^^26^^,^^27^ Besides, AFP remains the most widely adopted serologic marker for disease surveillance, with re-expression observed in 70%–80% of patients. Elevated AFP levels have been consistently correlated with advanced disease and unfavorable clinical outcomes, regardless of treatment modality.^28^^,^^29^

Beyond its role as a prognostic marker, AFP also contributes mechanistically to immune escape. Extracellular AFP can induce apoptosis in immune effector cells and dampen their cytotoxic function, while both intra- and extracellular AFP promote immune suppression by upregulating inhibitory molecules and tumor-associated antigens.^30^ Specifically, AFP disrupts immune equilibrium by altering CD4^+^/CD8^+^ T cell ratios and inhibiting the activity of natural killer cells, T lymphocytes, and dendritic cells.^31^ These effects collectively foster an immunosuppressive tumor microenvironment that undermines ICI efficacy.

Substantial clinical evidence further underscores AFP’s predictive relevance in the immunotherapy context. The REACH-2 trial, along with multiple retrospective studies, has identified baseline AFP as a significant predictor of response to ICI-based treatment.^32^ However, AFP expression is heterogeneous—approximately 40% of HCC patients, particularly those with early-stage disease, do not exhibit elevated AFP levels, while some advanced-stage cases may paradoxically show normal levels. This interpatient variability limits AFP’s standalone predictive power and underscores the need for composite biomarkers.

CRP, a canonical acute-phase reactant, reflects systemic inflammatory burden and has also been implicated in immunotherapy resistance.^24^ Elevated CRP levels have been shown to skew CD4^+^ T cell polarization toward a Th2 phenotype, impairing antitumor immune responses and diminishing ICI efficacy.^33^ Clinical studies have consistently demonstrated an association between high pretreatment CRP and shorter PFS and OS in HCC patients treated with ICIs.^12^ However, CRP is inherently nonspecific and may be elevated in diverse inflammatory, infectious, and neoplastic conditions, thereby limiting its standalone prognostic accuracy. As such, reliance on CRP in isolation may yield limited prognostic precision in HCC; its interpretation should be contextualized with complementary biomarkers for more robust clinical decision-making.

The integration of AFP and CRP into the CRAFITY score provides a mechanistically informed and clinically accessible framework that captures both tumor-intrinsic characteristics and systemic host immune status. While AFP reflects tumor burden and immune evasion, CRP serves as a proxy for systemic inflammation—two critical biological dimensions that impact immunotherapy responsiveness. Our findings align with the theoretical rationale of the CRAFITY score, demonstrating that higher baseline scores are strongly associated with inferior outcomes in patients receiving ICI-based therapy. This supports the clinical utility of the CRAFITY score as a composite biomarker for risk stratification and treatment guidance in HCC.

When considering prognostic assessment in HCC, the CRAFITY score should be evaluated in the context of existing markers such as the Child-Pugh score and BCLC staging. While Child-Pugh and BCLC remain standard tools for assessing liver function and tumor burden, respectively, the CRAFITY score provides complementary information by integrating systemic inflammation (via CRP) and liver function reserve (via AFP). This dual focus may allow the CRAFITY score to capture aspects of host-tumor interactions not fully addressed by conventional systems. Nonetheless, it should not be regarded as a replacement but rather as an adjunct to established prognostic models, particularly in patients undergoing immunotherapy where systemic inflammation may play a pivotal role.

Regarding heterogeneity observed in our meta-analysis, further sensitivity and subgroup analyses were performed. Variations in study region, treatment regimens (monotherapy vs. combination therapy), and patient baseline characteristics emerged as potential contributors. While the overall prognostic association between higher CRAFITY scores and poorer outcomes remained robust across subgroups, these findings underscore the importance of standardized study designs and patient selection in future research to minimize heterogeneity and strengthen external validity.

### Conclusion

In conclusion, our integrated analysis indicates that the CRAFITY score, which combines CRP and AFP levels, is associated with clinical outcomes in HCC patients undergoing ICI therapy. By reflecting both tumor-related and host inflammatory parameters, the score provides additional insights into the immunologic milieu that may influence treatment response. These findings suggest that the CRAFITY score holds promise as a complementary tool for risk stratification and individualized treatment planning. Nevertheless, its prognostic utility requires confirmation in future large-scale, prospective studies across diverse populations and treatment settings to establish its broader clinical applicability.

### Limitations of the study

Nonetheless, several limitations warrant consideration. First, both the meta-analysis and the single-center validation cohort are based on retrospective data, which may introduce bias and limit causal inference. The relatively small sample size of the single-center study could further contribute to potential selection bias, thereby restricting the generalizability of our findings. Second, the majority of studies included in the meta-analysis originated from East Asian populations, particularly China and Japan, which may constrain the applicability of the results to more diverse patient cohorts. Additionally, heterogeneity in treatment regimens and baseline characteristics across studies could have influenced the observed associations. To enhance the external validity of the CRAFITY score, future research should prioritize large-scale, multicenter, prospective studies employing standardized protocols across heterogeneous populations. Such efforts would be crucial to confirm the prognostic value of the CRAFITY score and to solidify its role as a reliable tool for risk stratification and clinical decision-making in the global immuno-oncology setting.

## Resource availability

### Lead contact

Further information and requests for resources and data should be directed to and will be fulfilled by the lead contact, Prof. Weixing Wang (wangwx@whu.edu.cn).

### Materials availability

This study did not generate new unique reagents, materials, or biological specimens.

### Data and code availability

• All data reported in this paper will be shared by the lead contact upon reasonable request.
• This paper does not report original code.
• Any additional information required to reanalyze the data reported in this paper is available from the lead contact upon request.

## Acknowledgments

None.

## Author contributions

L.Z., Y.Z., X.L., and W.W. conceptualized and designed the study. L.Z., Y.Z., J.F., C.C., X.L., and W.W. were responsible for data collection, assembly, analysis, and interpretation. Additionally, they contributed to manuscript drafting and were actively involved in its revision.

## Declaration of interests

The authors declare no competing interests.

## STAR★Methods

### Key resources table

**Table:** 

REAGENT or RESOURCE	SOURCE	IDENTIFIER
**Deposited data**		

PubMed	https://pubmed.ncbi.nlm.nih.gov/	N/A
EMBASE	https://www.embase.com/	N/A
The Cochrane Library	https://www.cochranelibrary.com/library	N/A

**Software and algorithms**		

Stata software Version 18.0	Downloaded STATA software	https://www.stata.com/products/

### Experimental model and study participant details

#### Human participants (retrospective cohort study)

This retrospective study included 129 consecutive patients diagnosed with HCC and treated with ICIs plus anti-angiogenic agents at Renmin Hospital of Wuhan University between June 2018 and July 2025.

#### Ethics approval and consent

The study was approved by the Ethics Committee of Renmin Hospital of Wuhan University. Written informed consent was waived due to the retrospective design, in accordance with institutional and international guidelines.

#### Demographic and clinical characteristics

The median age was 56.4 years (range: 38.5–81.1), and 83 patients (64.3%) were male. Baseline characteristics including ECOG performance status, BCLC stage, Child–Pugh class, and presence of cirrhosis are provided in Table S1.

#### Eligibility and exclusion criteria

Eligible patients were required to have at least one radiologically measurable lesion (RECIST v1.1) and confirmed diagnosis of HCC by AASLD criteria.^34^ Exclusion criteria included: (i) missing baseline AFP or CRP data; (ii) elevated CRP due to infection or systemic inflammation; (iii) concurrent malignancies other than HCC; (iv) Child–Pugh C liver function; and (v) BCLC stage D disease.

### Method details

#### Data extraction and quality assessment

Two investigators independently extracted data, including study design, population characteristics (age, sex), treatment regimen, follow-up duration, and outcome measures. When both multivariable and univariable HRs were available, those derived from multivariate models were preferentially included to ensure robustness of effect estimates.^35^ Discrepancies were resolved by consensus with a senior investigator. Study quality was assessed using the Newcastle–Ottawa Scale (NOS), with scores >6 considered high quality.

#### Retrospective clinical data collection

Electronic medical records were reviewed to extract demographics, viral hepatitis status, Child–Pugh classification, ALBI grade, tumor burden, and treatment line. Tumor response was assessed every 1–3 months using contrast-enhanced CT or MRI following RECIST v1.1 criteria.

#### CRAFITY score definition

Patients were categorized as: CRAFITY-low: AFP < 100 ng/mL and CRP < 10 mg/dL; CRAFITY-intermediate: one parameter elevated; CRAFITY-high: both parameters elevated.

#### Meta-analysis study selection

A systematic literature search was performed in PubMed, EMBASE, and the Cochrane Library (cut-off date: June 2025) using the keyword “CRAFITY.” Grey literature from Google Scholar and the reference lists of eligible studies was also screened. Studies were included if they (1) enrolled HCC patients; (2) evaluated ICIs as treatment; (3) reported OS, PFS, ORR, or DCR stratified by CRAFITY score; and (4) provided sufficient data to estimate HRs or odds ratios (ORs). Conference abstracts, reviews, editorials, and studies with overlapping cohorts were excluded; the most comprehensive dataset was retained when duplicates were identified.

### Quantification and statistical analysis

Categorical variables are presented as counts and percentages. Continuous variables are reported as median (range). OS and PFS were estimated using Kaplan–Meier curves and compared by log-rank test.

Meta-analyses were performed in Stata (v18.0) using random-effects (DerSimonian–Laird) or fixed-effects models depending on heterogeneity (I^2^ > 50% or Cochran’s Q *p* < 0.1 indicating substantial heterogeneity).^36^ Sensitivity analyses were performed by sequential exclusion of studies. Publication bias was assessed by Begg’s and Egger’s tests and visual inspection of funnel plots.

All p-values were two-sided, and *p* < 0.05 was considered statistically significant. Statistical details and definitions of center and dispersion (median, 95% CI) are reported in the figure legends and results section.

### Additional resources

No additional resources were generated for this study.
