# Supplementary material for: Prognostic impact of CRAFITY score in hepatocellular carcinoma patients treated with immune checkpoint inhibitors
Source: iScience. 2025 Nov 10;28(12):113976. doi: 10.1016/j.isci.2025.113976 (PMC12677073; doi:10.1016/j.isci.2025.113976)
Supplement: Document S1. Figures S1–S6 and Table S1 [file mmc1.pdf]

iScience, Volume *28*

Supplemental information

Prognostic impact of CRAFITY score in hepatocellular carcinoma patients treated with immune checkpoint inhibitors

Lilong Zhang, Yuefeng Zhang, Kunpeng Wang, Jiarui Feng, Chen Chen, Xinfei Liu, and Weixing Wang

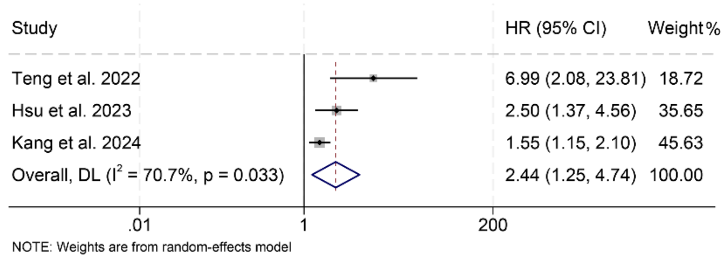

Fig. S1. Forest plot analyses illustrating the correlation between initial CRAFITY score and overall survival outcomes in hepatocellular carcinoma patients undergoing immune checkpoint inhibitor therapy.

Abbreviations: HR, hazard ratio; CI, confidence interval.

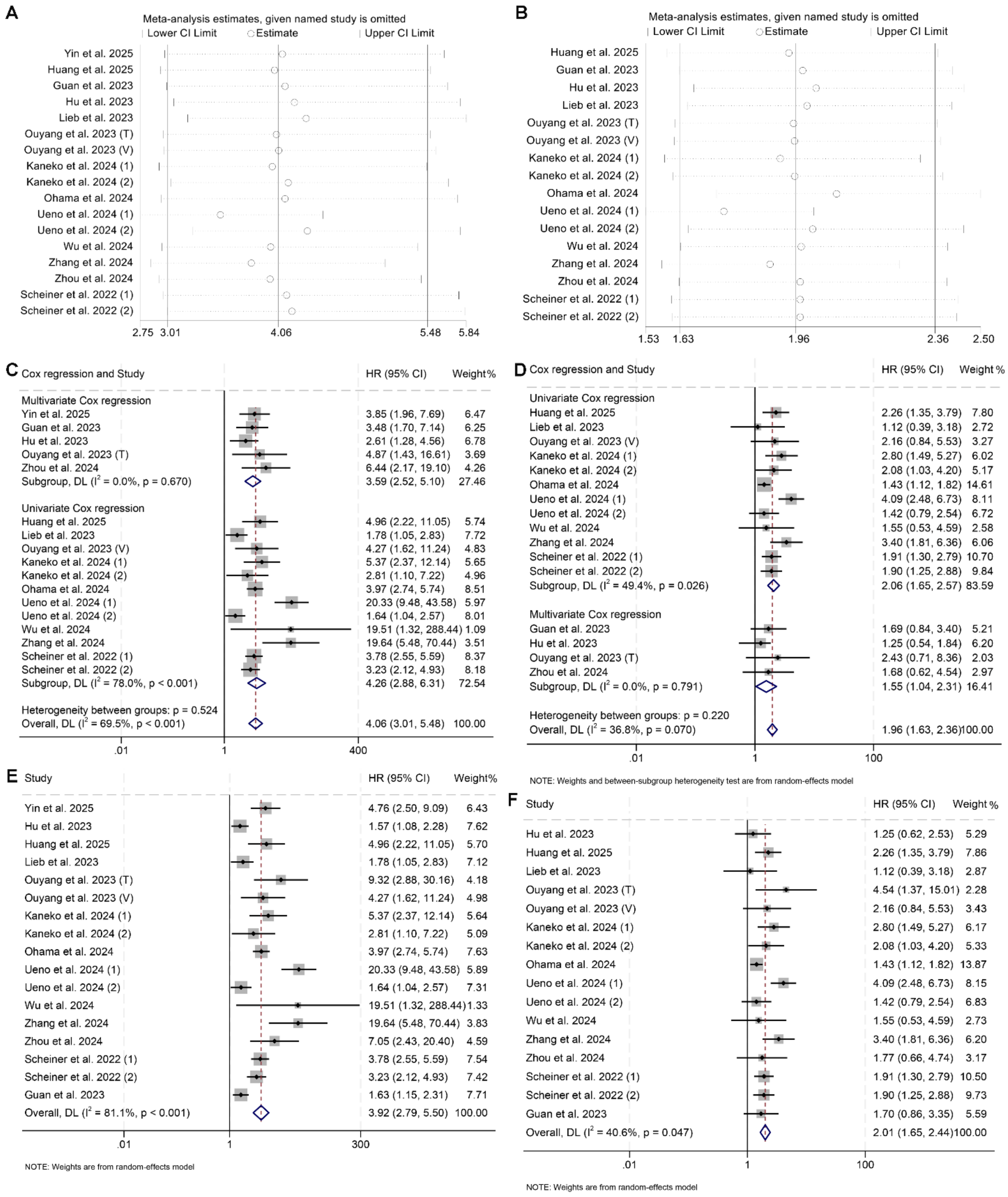

Fig. S2. Sensitivity and stratified analyses demonstrate the association between baseline CRAFITY classification and overall survival in hepatocellular carcinoma patients treated with immune checkpoint inhibitors.

(A) Sensitivity analysis comparing high vs. low CRAFITY score groups. (B) Sensitivity analysis comparing intermediate vs. low CRAFITY score groups.(C) Stratified analysis using Cox proportional hazards models comparing high vs. low CRAFITY scores. (D) Stratified analysis using Cox proportional hazards models comparing intermediate vs. low CRAFITY scores. (E) Forest plot of univariate model evaluating high vs. low CRAFITY scores. (F) Forest plot of univariate model evaluating intermediate vs. low CRAFITY scores.

Abbreviations: HR, hazard ratio; CI, confidence interval.

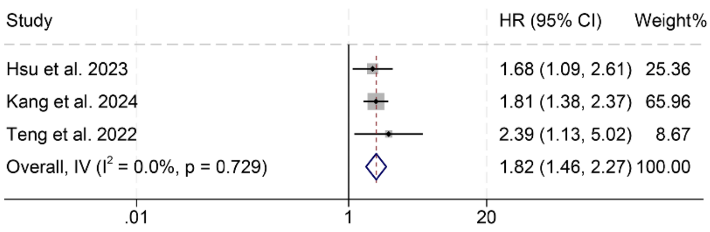

Fig. S3. Forest plot analyses illustrating the correlation between initial CRAFITY score and progression-free survival in hepatocellular carcinoma patients undergoing immune checkpoint inhibitor therapy.

Abbreviations: HR, hazard ratio; CI, confidence interval.

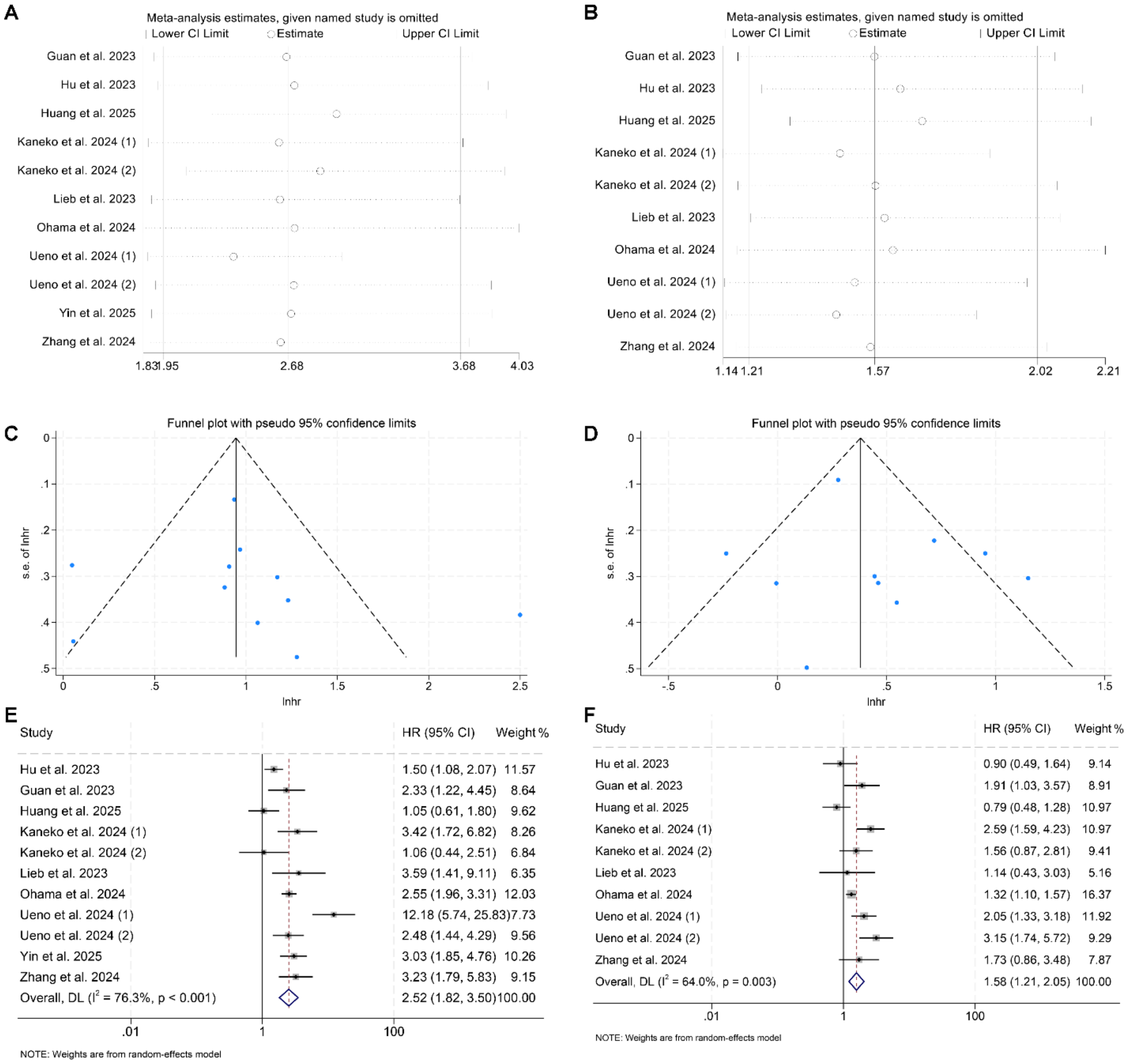

Fig. S4. Sensitivity, funnel, and univariate analyses reveal the relationship between CRAFITY classification and progression-free survival in hepatocellular carcinoma patients treated with immune checkpoint inhibitors.

(A) Sensitivity analysis comparing high vs. low CRAFITY score groups. (B) Sensitivity analysis comparing intermediate vs. low CRAFITY score groups. (C) Funnel plot assessing publication bias for high vs. low CRAFITY scores. (D) Funnel plot assessing publication bias for intermediate vs. low CRAFITY scores. (E) Forest plot based on

univariate models comparing high vs. low CRAFITY scores. (F) Forest plot based on univariate models comparing intermediate vs. low CRAFITY scores.

Abbreviations: HR, hazard ratio; CI, confidence interval.

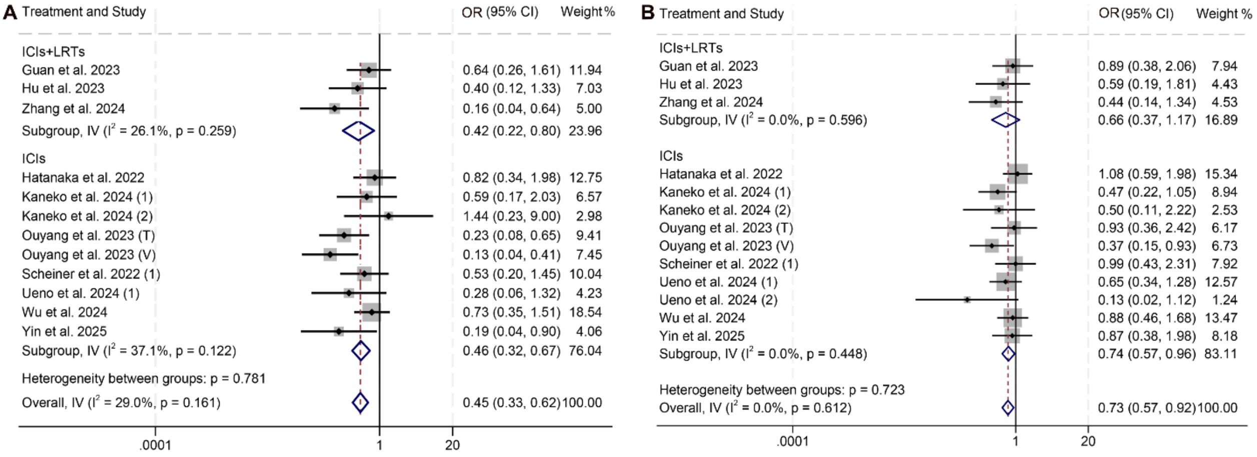

Fig. S5. Stratified analyses according to treatment modality demonstrate the prognostic impact of CRAFITY classification on objective response rate in hepatocellular carcinoma patients.

(A) Comparison of high vs. low CRAFITY scores. (B) Comparison of intermediate vs. low CRAFITY scores.

Abbreviations: OR, odds ratio; CI, confidence interval.

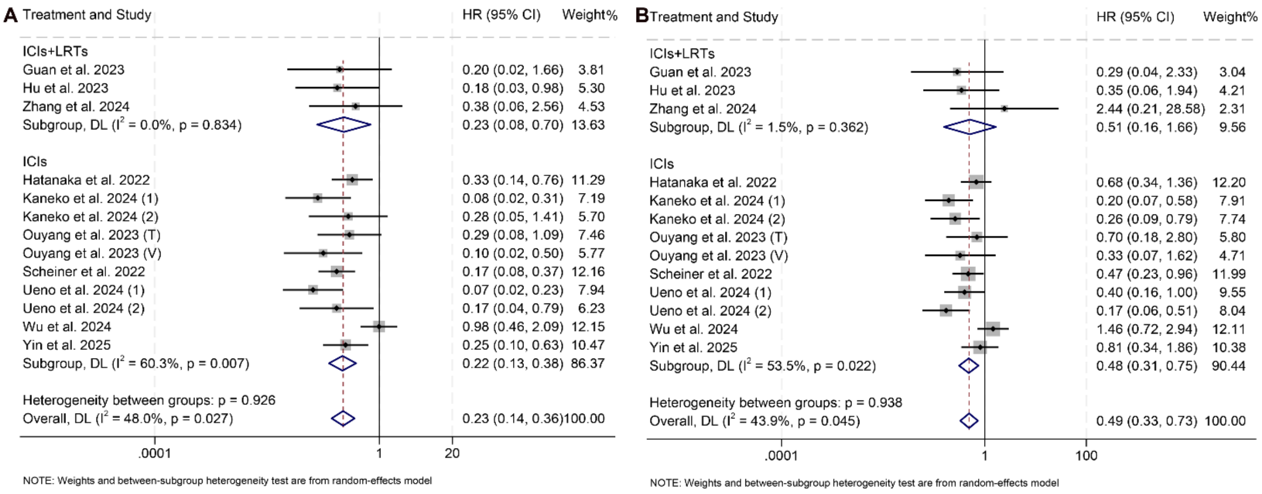

Figure S6. Stratified analyses according to treatment modality demonstrate the prognostic impact of CRAFITY classification on disease control rate in hepatocellular carcinoma patients.

(A) Comparison of high vs. low CRAFITY scores. (B) Comparison of intermediate vs. low CRAFITY scores.

Supplementary Table 1. Patient characteristics

	Overall (n=129)
Age	56.4 (38.5-81.1)
Males	83 (64.34%)
ECOG PS	
0	85 (65.89%)
1	44 (34.11%)
Etiology	
Viral	102 (79.07%)
Other	27 (20.93%)
Liver cirrhosis	
Yes	96 (74.42%)
No	33 (25.58%)
BCLC stage	
Early	7 (5.43%)
Intermediate	37 (28.68%)
Advanced	85 (65.89%)
Child-Pugh class	
A	76 (58.91%)
B	53 (41.09%)
Tumor number	
< 3	69 (53.49%)
≥ 3	60 (46.51%)
Macrovascular invasion	
Yes	62 (48.06%)
No	67 (51.94%)
Treatment line	
First-line	98 (75.97%)
Later-line	31 (24.03%)
ALBI grade	
1	58 (44.96%)
2	71 (55.04%)

Data shown are means with range or numbers with percentage.

ECOG PS, Eastern Cooperative Oncology Group performance status; BCLC, Barcelona Clinic Liver Cancer; mALBI grade, modified albumin-bilirubin grade.
